# Repeat Upper Gastrointestinal Endoscopy in Patients with Functional Dyspepsia: Yield, Findings, and Predictors of Positive Findings

**DOI:** 10.1155/2015/904683

**Published:** 2015-04-12

**Authors:** Supot Pongprasobchai, Natta Asanaleykha, Pongchirat Tantayakom

**Affiliations:** Division of Gastroenterology, Department of Medicine, Faculty of Medicine, Siriraj Hospital, Mahidol University, Bangkok 10120, Thailand

## Abstract

*Background*. No guideline on repeat esophagogastroduodenoscopy (EGD) in functional dyspepsia (FD) exists. This study aimed to define yield, findings, and predictors of positive findings on repeat EGD in FD. *Methods*. FD patients who underwent at least 2 EGDs during October 2005 to November 2011 were enrolled and reviewed. Yield and findings were analyzed and univariate and multivariate analyses were performed to identify predictors of positive repeat EGD. *Results*. The median time to repeat EGD was 34 months. Among 146 patients, 115 patients (79%) had negative and 31 (21%) had positive repeat EGD, including erosive gastritis (13.0%), peptic ulcer (7.5%), reflux esophagitis (1.4%), and Barrett's esophagus (0.7%). Four independent predictors of positive repeat EGD were smoking (HR 3.88, 95% CI 1.31–11.51, *P* = 0.015), hypertension (HR 2.96, 95% CI 1.38–6.36, *P* = 0.050), history of malignancies (HR 3.65, 95% CI 1.16–11.46, *P* = 0.027), and antiplatelets or NSAIDs used within 4 weeks (HR 4.10, 95% CI 1.13–14.90, *P* = 0.032), while alarm features or failure to treatment did not predict positive repeat EGD.  *Conclusion*. Yield of repeat EGD in FD was substantially low, all findings were acid-related disorders, and there was no malignancy. Smoking, hypertension, history of malignancies, and antiplatelets/NSAIDs use associated with positive repeat EGD.

## 1. Introduction

Dyspepsia is the most common gastrointestinal problem in general practice, occurring in 10–50% of the population each year [1, 2]. Of all types of dyspepsia, functional dyspepsia (FD) is the most common (70–90%) [3], while organic dyspepsia is found in only a minority of patients. Thus, many guidelines including Thailand's recommend performing esophagogastroduodenoscopy (EGD) to only dyspeptic patients who are older than 55 years old or having alarm features [4, 5] in order to reduce the number of patients finally having normal or trivial findings on EGD, which is FD.

The current treatment of FD remains disappointing [6–8]. Patients usually run a chronic course with alternation between improvement and exacerbation. Many FD patients eventually undergo repeat EGD due to the chronicity of the symptoms, the refractoriness to treatment, the presence of new alarm symptoms, patient anxiety, or even the doctor's own fear of misdiagnosis. Currently, the evidence on the yield, findings of repeat EGD, and the clinical parameters to predict patients who are likely to have positive significant findings on repeat EGD are still lacking. Only few studies have been reported but showed conflicting results [9, 10]. There is no consensus guideline on the optimal indications for repeating EGD in FD [4, 5]. Thus, the aim of this study is to evaluate the frequency, reasons for repeating EGD, findings, and predictors of positive findings in patients with FD in order to help physicians select more appropriate patients for repeat EGD in the future.

## 2. Methods

### 2.1. Study Population

This study was approved by the Siriraj Institutional Review Board. The study site was Siriraj Hospital, a tertiary care university hospital in Bangkok, Thailand. All consecutive patients who presented with dyspepsia and had undergone at least 2 EGDs in our hospital during October 2005 to November 2011 were enrolled.

### 2.2. Endoscopic Database and Search Strategy

The endoscopic database was searched systematically to identify all patients with FD who underwent repeat EGD for the evaluation of dyspepsia. Patients with dyspepsia were identified by searching the terms “dyspepsia,” “epigastric pain,” or “abdominal pain” in the “indications” field. Patients who underwent at least 2 EGDs with an indication of dyspepsia were included. The inclusion criteria were as follows: (1) patients with FD, defined by ROME III criteria [11], (2) age >18 years, (3) patients who underwent at least 2 EGDs, (4) the first EGD showed normal finding, nonerosive gastritis, or any lesion that could not explain the symptom of dyspepsia [12]. For all studies in which biopsies were performed, the histological diagnoses were confirmed by reviewing of electronic pathology records.

### 2.3. Data Collection and Definitions

Data were extracted from the medical records, endoscopic and pathological reports. Demographic data included gender, age, comorbid diseases, history of smoking and alcohol drinking, history of gastrointestinal malignancy in first degree relatives, subtype of FD, that is, postprandial distress syndrome (PDS), epigastric pain syndrome (EPS), or mixed subtype, duration of dyspepsia before the first and second EGD, alarm symptoms, night pain/awakening pain, and history of specific drug used within 4 weeks, for example, antiplatelets, aspirin, nonsteroidal anti-inflammatory drugs (NSAIDs), corticosteroid, and proton pump inhibitors (PPI).

Endoscopic data included procedure date, indication of repeat EGD, endoscopic findings, and* Helicobacter pylori *status. Findings of the EGD were categorized as positive if there were erosive gastritis, peptic ulcer, reflux esophagitis, Barrett's esophagus, or malignancy and categorized as negative when they were normal, nonerosive gastritis or revealed no evidence of structural disease that likely explained the symptoms [12].

### 2.4. Factors Associated with Positive Repeat EGD

Data of patients with positive and negative repeat EGD were compared using univariate and multivariate analyses.

### 2.5. Statistical Analysis

Statistical analysis was done by using SPSS Program version 17.0. Yield and findings were calculated using descriptive statistics and presented with number and percent. The associations between clinical parameters and the results of repeat upper endoscopy used Chi-square test or Fisher-exact test for categorical variables and Student's *t*-test or Mann-Whitney *U* test for continuous variable data. Variables were considered significant when *P* value was <0.05 in univariate analyses and logistic regression analysis was performed to identify independent factors associated with positive repeat EGD and presented with hazard ratio and 95% confidence interval. Statistical significance was considered when *P* value was <0.05.

## 3. Results

### 3.1. Study Population

**Figure 1:**
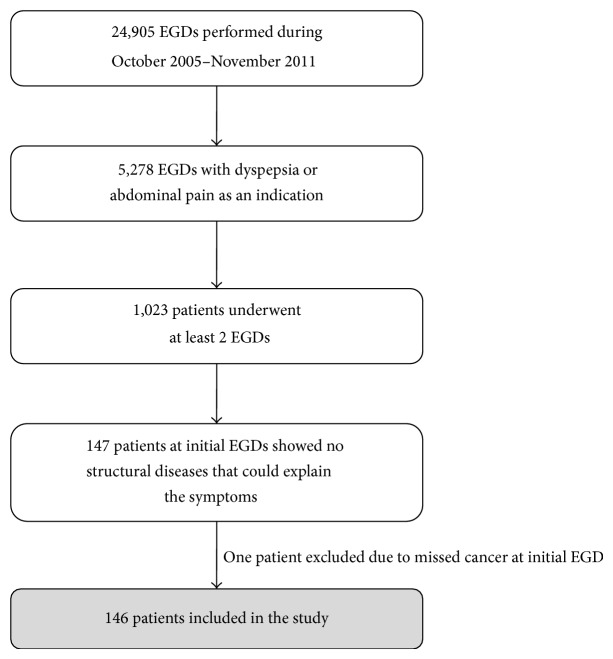
Study population (EGD, esophagogastroduodenoscopy).

During the study period, a total of 24,905 EGDs were performed in our institute. Of these, 5,278 (21.2%) had dyspepsia or abdominal pain as indications for EGD. There were 1,023 patients (19.4%) who underwent at least 2 EGDs for the evaluation of dyspepsia or abdominal pain, of which 146 (14.3%) had FD at the initial EGDs.* Helicobacter pylori* was tested in all cases and was positive in 25 patients (17%), of which* H. pylori* eradication was done in all. One patient was diagnosed as FD during the initial EGD, which showed chronic gastritis. However, the subsequent second EGD done 1 year later revealed linitis plastica from gastric adenocarcinoma. On the review, the first EGD pictures already had rugal thickening suggestive of linitis plastica but the mucosal biopsy was negative. Thus, this patient was considered a misdiagnosis and not included in the study (Figure 1).

### 3.2. Demographic Data

The demographic data and procedure-related characteristics of the study patients are summarized in Table 1. The mean age was 56.8 ± 11.6 years and 63.7% were female. Twelve patients (8.2%) drank alcohol and 13 patients (8.9%) smoked. Eighty patients (54.8%) had comorbid diseases including diabetic mellitus (18 patients, 12.3%), essential hypertension (58 patients, 39.7%), dyslipidemia (43 patients, 29.5%), cardiovascular disease (5 patients, 3.4%), coronary artery disease (12 patients, 8.2%), kidney disease (1 patient, 0.7%), and malignancy (7 patients, 4.8%). Seven patients (4.8%) had gastrointestinal malignancies in their first degree relatives.

### 3.3. Clinical Features, Duration to Repeat EGD, and Indications

The clinical features of dyspepsia during the repeat EGD were EPS (104 patients, 71.2%), PDS (34 patients, 23.3%), and mixed subtype (1 patient, 0.7%) and were not defined in 7 patients (4.8%). Twenty-nine patients (19.9%) had alarm features such as unexplained weight loss (21 patients, 14.4%) and gastrointestinal blood loss (8 patients, 5.5%). Night pain or awakening pain was found in 4 patients (2.7%) and history of aspirin or NSAID used within 4 weeks was found in 34 patients (23.3%).

Repeat EGD was performed at a median of 34.0 months (IQR, 1–168 months) after initial EGD. The indications for repeat EGD are shown in Table 1. Most indications are dyspepsia with failed medication therapy (50.7%) followed by dyspepsia with alarm features (20.6%), dyspepsia with age ≥55 years (8.2%), patients' request (6.2%), and others 8.9%.

### 3.4. Yield and Findings of Repeat EGD

Findings of the repeat EGD are shown in Table 2. Thirty-one patients (21.2%) had positive findings, which were erosive gastritis (13.0%), peptic ulcer (7.5%), reflux esophagitis (1.4%), and Barrett's esophagus (0.7%). Negative finding was found in 115 patients (78.8%).* H. pylori* was tested in all cases and was positive in 9 patients (6.2%).

### 3.5. Factors Associated with Positive Repeat EGD

Data of 115 patients with negative findings and 31 patients who had positive findings on repeat EGD were compared using univariate analyses (Table 3). Demographic data were almost similar except more comorbid illnesses of hypertension (58.1 versus 34.8%, *P* = 0.019) and malignancies (12.9% versus 2.6%, *P* = 0.037) in the positive than in the negative repeat EGD group, respectively. The median times of repeat EGD were similar. There were more antiplatelets or NSAIDs used in the positive repeat EGD group (36% versus 20%, *P* = 0.007). The indications of repeat EGD, the presence of alarm features,* H. pylori* status, and prior prescription of PPI were not different.

Multivariate analysis was performed (Table 3). Four factors were found to be independent factors associated with positive findings on repeat EGD. They were smoking (HR 3.88, 95% CI 1.31–11.51, *P* = 0.015), comorbid disease of hypertension (HR 2.96, 95% CI 1.38–6.36, *P* = 0.050), history of malignancies (HR 3.65, 95% CI 1.16–11.46, *P* = 0.027), and history of antiplatelets or NSAIDs used within 4 weeks (HR 4.10, 95% CI 1.13–14.90, *P* = 0.032). Details of the positive repeat EGD in patients with these 4 factors are shown in Table 4.

## 4. Discussion

FD is a chronic functional gastroduodenal disorder characterized by its remitting and exacerbating nature. This may lead patients to the repeat EGD due to the fear of serious diseases, by either the patients or the physicians. Currently, there is no recommendation on the optimal indications and timing for repeating EGD.

In the present study, the authors demonstrated that 14% of the patients who had FD underwent repeat EGD. It has been estimated from the randomized trials on the management strategies for dyspepsia that the rates of repeat EGD were 5–25% in 1 year [13–16] and 9–26% in 6-7 years [17, 18]. The 14% rate of repeat EGD in the present study, although it was comparable to the rates above, might be underestimated because some patients might had repeat EGD at the hospitals outside our institute. Longer-term follow-up of patients with FD in the randomized trials showed that most of the repeat EGD was within the initial year of the studies [13, 17, 18]. In the present study, the median duration of repeat EGD was 34 months (IQR, 1–168 months) after the initial EGD, which is slightly later than previous studies. The reason is unknown but from our perception we postulate that Asian patients are more easily assured after having negative EGD and then being informed that they have FD; dyspeptic symptoms often decline after EGD. Therefore, repeat EGD might be more delayed than in the Westerners.

Yield of positive significant lesions on the repeat EGD in the present study was 21%. All of the findings were acid-related disorders and no malignancy was found during the median of 34 months. Data in the literature is limited regarding the yield of repeat EGD in FD. Result of the present study showing 21% positive significant findings is close to the results of the previous studies by Ladabaum and Dinh (18%) [9] and close to the 15–20% rate of the significant lesions by an initial EGD from the systematic review [19]. Although there is a recent study by Guo et al. [10] showing the much lower rate of significant lesions during repeat EGD of FD (0.5%), the yield was, anyway, similar to initial EGD in their study (0%) [10]. The only difference is that malignancy usually accounts for approximately 1% of the initial EGD [19] but has never been found in all studies of repeat EGD [9, 19], including the present study.

The present study is the first study to evaluate the predictors for positive findings on repeat EGD. The results might help physicians select patients who will likely derive benefit from repeat EGD. The present study demonstrated 4 factors that independently associated with positive findings on repeat EGD, that is, smoking, hypertension, malignancies, and history of antiplatelets/NSAIDs used within 4 weeks. On the other hand, the presence of alarm features, failure to respond to medical therapy, or the* H. pylori *status did not associate with positive repeat EGD.

Cigarette smoking, antiplatelets, and NSAIDs are the well-known risk factors for peptic ulcer diseases [20]. Smoking increases gastric acid secretion by the stimulation of histamine released after mast cell degranulation and from the increased functional parietal cell volume [21, 22]. Aspirin and NSAIDs can induce peptic ulcers via inhibiting of the cyclooxygenase (COX) pathways. Interestingly, the present study demonstrated that comorbid diseases, that is, hypertension and the presence of malignancies, increased the risk of positive findings on repeat EGD. The explanations for these relationships are unclear. Patients with known malignancies may undergo chemotherapy, radiation and may be exposed to many medications, for example, NSAIDs or serious illness (the risk for stress-related mucosal disease). Furthermore, physicians were probably more alert when these patients had dyspepsia, causing more frequent work-ups. Unfortunately, the present study did not have information to verify these postulations. Similarly, the reason why the patient with hypertension was associated with positive repeat EGD (i.e., peptic ulcers and erosive gastritis) is also difficult to explain, but it is possible that some patients might receive aspirin for coronary artery disease prophylaxis. Thus, further study is needed to clarify the reasons beneath these associations.

The present study found that the presence of alarm features, failure to respond to medical therapy of FD, and* H. pylori *status, which sound sensible to have positive EGD, turned out to be unrelated to the positive repeat EGD. This is not surprising because meta-analysis has shown only a very limited predictive value of alarm features in patients with dyspepsia [23] and it is quite common for FD to be unresponsive to medical therapy because current treatment remains unsatisfactory [6–8]. Thus, the presence of alarm features or failure of treatment does not predict positive repeat EGD. Therefore, should we avoid repeating EGD in FD patients who have alarm features or do not respond to treatment? The answer is probably no, because missed or newly developed upper gastrointestinal cancers after negative initial EGD have been reported to be around 6% [24]. However, most patients (around 3 in 4) with missed esophageal or gastric cancers found within 3 years after the initial EGD usually had alarm symptoms at presentations and the initial EGD usually had some suspicious findings but physicians might not pay enough attention to prove them [24, 25]. The present study also found one case of missed linitis plastica since it was overlooked during initial EGD. Therefore, it is the authors' personal suggestion that we may repeat EGD in patient with alarm symptoms or failed treatment when the result of initial EGD looks inadequate or incomplete or no official endoscopic report is available.

*H. pylori* is an important factor that should be associated with the positive repeat EGD, particularly peptic ulcer disease and erosive gastritis, which were the 2 most common findings of the positive EGD group. Although the present study showed the low and indifferent rates of* H. pylori* among the positive and negative EGD groups, it remains difficult to conclude that* H. pylori *had no role in the presence of positive EGD because most patients (77%) in the present study had PPI therapy within 8 weeks before the repeat EGD. Thus, it might cause significant false-negative* H. pylori *testing in the present study.

There are some limitations of the present study. First, because it was a single-center retrospective study, some repeat EGD at other hospitals might be missed and not included. Second, the decision to repeat EGD was on-demand and depended on the attending physicians, not to every patient in a certain interval. Thus, the frequency of positive repeat EGD in the present study might not represent the real frequency in FD. However, even in these highly selected patients, the yield of repeat EGD remained low. Third, we used “dyspepsia” and “abdominal pain” for the indication to perform EGD as the searching keywords; thus we might miss some patients with FD that might use other words. However, we believe that these words are proper enough for indicating dyspepsia.

## 5. Conclusions

The yield and findings of repeat EGD in Thai patients with FD were substantially low; most findings were minor acid-related disorders and no malignancy was found during the median 3-year follow-up. Cigarette smoking, hypertension, history of malignancies, and history of antiplatelets or NSAIDs used within 4 weeks were associated with positive findings on repeat EGD.

## Figures and Tables

**Table 1:** Demographic characteristics of the 146 patients.

Characteristics	Number (%) or mean ± SD
Age (years), mean ± SD	56.8 ± 11.6
Gender (female), *n* (%)	93 (63.7)
Time from the first EGD to repeat EGD (months), median (range)	34 (1–168)
Indication of repeat EGD, *n* (%)	
Dyspepsia with age ≥55 years	12 (8.2)
Dyspepsia with alarm features	30 (20.6)
Dyspepsia with failed medical therapy	74 (50.7)
Patients' request	9 (6.2)
Others	13 (8.9)
Not specified	5 (3.4)

EGD, esophagogastroduodenoscopy; SD, standard deviation.

**Table 2:** Findings and diagnosis of the repeat EGD in 146 patients.

Findings	Number (%)
Negative	115 (78.8)
Positive	31 (21.2)
Erosive gastritis	19 (13.0)
Peptic ulcer	11 (7.5)
Gastric ulcer	6 (4.1)
Duodenal ulcer	5 (3.4)
Reflux esophagitis	2 (1.4)
Barrett's esophagus	1 (0.7)

**Table 3:** Univariate and multivariate analyses of the clinical parameters between patients with positive and negative findings on repeat EGD (data are shown in *n* (%), unless specified).

Clinical parameters	Univariate analysis			Multivariate analysis	
	Negative repeat EGD (*n* = 115)	Positive repeat EGD (*n* = 31)	*P*	Hazard ratio (95% CI)	*P*
Age (years), mean ± SD	56.4 ± 11.8	58.3 ± 10.9	0.432		
Gender, female	75 (65.2)	18 (58.1)	0.462		
Time to repeat EGD (months), median (range)	33.5 (1–168)	36.1 (7–118)	0.421		
Comorbid illnesses					
Diabetes	15 (13.0)	3 (9.7)	0.765		
Hypertension	40 (34.8)	18 (58.1)	0.019	2.96 (1.38–6.36)	0.050
Dyslipidemia	32 (27.8)	11 (35.5)	0.406		
Coronary artery disease	7 (6.1)	5 (16.1)	0.071		
Kidney diseases	1 (0.9)	0	1.000		
Liver diseases	0	0	1.000		
Malignancies	3 (2.6)	4 (12.9)	0.037	3.65 (1.16–11.46)	0.027
Family history of GI malignancies	6 (5.2)	1 (3.2)	1.000		
Smoking	8 (6.9)	5 (16.1)	0.015	3.88 (1.31–11.51)	0.015
Alcohol drinking	11 (9.5)	1 (3.2)	0.462		
Use of NSAIDs or antiplatelets	23 (20.0)	11 (35.5)	0.007	4.10 (1.13–14.90)	0.032
Night pain/awakening pain	3 (2.6)	1 (3.2)	1.000		
Indications of repeat EGD					
Dyspepsia with age ≥55 years	11 (9.5)	1 (3.2)	0.462		
Dyspepsia with alarm features	24 (20.9)	6 (19.4)	0.853		
Dyspepsia with failed medical therapy	56 (48.7)	18 (58.1)	0.354		
Patients' request	6 (5.2)	3 (9.7)	0.401		
Others	10 (8.7)	3 (9.7)	1.000		
Not specified	4 (3.5)	1 (3.2)	1.000		
Alarm features					
Dysphagia	0	0	—		
Unexplained weight loss	17 (14.8)	4 (12.9)	1.000		
Persistent vomiting	0	0	—		
Evidence of GI blood loss	7 (6.1)	1 (3.2)	1.000		
Use of PPI within 8 wk	89 (77.4)	23 (74.2)	0.709		
*H. pylori* infection	6 (5.2)	3 (9.7)	0.400		

EGD, esophagogastroduodenoscopy; GI, gastrointestinal; NSAIDs, non-steroidal anti-inflammatory drugs; PPI, proton pump inhibitor; SD, standard deviation.

**Table 4:** Details of the positive repeat EGD in patients with the 4 factors for positive repeat EGD.

Presence of predictors	*n*	Positive repeat EGD				Negative repeat EGD
		Peptic ulcer	Erosive gastritis	Reflux esophagitis	Others	
Hypertension	58	8	9	2		40
History of malignancies	7	1	3			3
Smoking	13	1	1	1	1	8
Use of NSAIDs or antiplatelets	34	4	9	1		23
